# The diagnostic potential of low-field MRI in problematic total knee arthroplasties - a feasibility study

**DOI:** 10.1186/s40634-020-00274-2

**Published:** 2020-07-31

**Authors:** Femke F. Schröder, Corine E. Post, Sjoerd M. van Raak, Frank F. J. Simonis, Frank-Christiaan B. M. Wagenaar, Rianne M. H. A. Huis in’t Veld, Nico Verdonschot

**Affiliations:** 1 OCON, centre for orthopaedic surgery, Geerdinksweg 141 postbus 546, 7550 AM Hengelo, The Netherlands; 2 University of Twente, Faculty of Engineering Technology, Biomechanical Engineering, postbus 217 7500 AE, Enschede, The Netherlands; 3 Orthopaedic Research Laboratory, Radboud University Medical Center, postbus, 9101 6500 HB Nijmegen, The Netherlands; 4 Department of Radiology, Ziekenhuisgroep Twente, Zilvermeeuw 1, 7609PP, Almelo, The Netherlands; 5 University of Twente, Faculty of science and technology, Magnetic Detection and Imaging, postbus 217 7500 AE, Enschede, The Netherlands

**Keywords:** Low field MRI, Weight-bearing MRI, Total knee arthroplasty, Problematic TKA

## Abstract

**Purpose:**

Low-field MRI, allowing imaging in supine and weight-bearing position, may be utilized as a non-invasive and affordable tool to differentiate between causes of dissatisfaction after TKA (‘problematic TKA’). However, it remains unclear whether low-field MRI results in sufficient image quality with limited metal artefacts. Therefore, this feasibility study explored the diagnostic value of low-field MRI concerning pathologies associated with problematic TKA’s’ by comparing low-field MRI findings with CT and surgical findings. Secondly, differences in patellofemoral parameters between supine and weight-bearing low-field MRI were evaluated.

**Methods:**

Eight patients with a problematic TKA were scanned using low-field MRI in weight-bearing and supine conditions. Six of these patients underwent revision surgery. Scans were analysed by a radiologist for pathologies associated with a problematic TKA. Additional patellofemoral and alignment parameters were measured by an imaging expert. MRI observations were compared to those obtained with CT, the diagnosis based on the clinical work-up, and findings during revision surgery.

**Results:**

MRI observations of rotational malalignment, component loosening and patellofemoral arthrosis were comparable with the clinical diagnosis (six out of eight) and were confirmed during surgery (four out of six). All MRI observations were in line with CT findings (seven out of seven). Clinical diagnosis and surgical findings of collateral excessive laxity could not be confirmed with MRI (two out of eight).

**Conclusion:**

Low-field MRI shows comparable diagnostic value as CT and might be a future low cost and ionizing radiation free alternative. Differences between supine and weight-bearing MRI did not yield clinically relevant information.

The study was approved by the Medical Research Ethics Committees of Twente (Netherlands Trial Register: Trial NL7009 (NTR7207). Registered 5 March 2018, https://www.trialregister.nl/trial/7009).

## Background

Total knee arthroplasty (TKA) is a highly successful procedure usually performed on patients with end-stage osteoarthritis to improve long-term function and reduce pain [1]. Each year, more than 700,000 TKA procedures are performed in the US, and that figure has been increasing annually [1, 2]. Despite the increase, approximately 20% of the patients are dissatisfied after TKA; these patients’ cases are referred to as the problematic TKA [1]. Pathologies related to this dissatisfaction include intra-articular, peri-articular and extra-articular causes. The classical described pathologies for which revision is performed include loosening, infection, instability, and malalignment [1, 3–6]. Medical imaging of the TKA plays an important role in identifying the cause(s) of dissatisfaction. During the past decade, several differential diagnostic algorithms for the problematic TKA have been developed as a result of a multitude of studies [3, 7–9]. In all these differential diagnostic algorithms several additional imaging investigations such as CT, SPECT-CT, stress radiographs or other are used. Unfortunately, none of these imaging techniques are solely able to diagnose all probable causes of the problematic TKA simultaneously.

In the native knee, MRI has become the standard to evaluate the joint and surrounding soft tissue [10]. MRI is considered to be of limited diagnostic value after TKA, primarily due to metallic susceptibility artefacts caused by the metal implant [11]. Recent review articles have described how it is possible to evaluate a TKA with MRI using optimized sequences and advanced metal artefact reduction techniques [2, 12]. However, despite these efforts, susceptibility artefacts are still present. Another method to reduce these artefacts is to decrease the main magnetic field, i.e. use low-field MRI [13]. Although low-field MRI (≤ 1 T) was previously regarded as having inferior imaging quality, systems have improved through the years [14, 15]. Together with the possibility to reduce susceptibility artefacts, low-field MRI is hypothesized to be a potential solution to evaluate the problematic TKA and its surrounding soft tissue.

The majority of medical imaging is performed with the patient in a supine position, except the conventional weight-bearing long-leg view. The knee is a dynamic joint acting in a load-bearing capacity during the day. Therefore, evaluation of the knee in a load-bearing situation may offer improved and more relevant insight in some pathologies. For example, in the native knee, deviated patellar height in the weight-bearing position might be associated with lateral displacement and patellar tilt [16]. Patellofemoral maltracking is considered to be diagnosed more effectively in the weight-bearing position [17], whereby the tibial tubercle-trochlear groove distance (TT-TG) has been reported to decrease [18]; whether this also applies after TKA is currently unknown.

Taken together, the absence of a single imaging technique that can simultaneously differentially diagnose a problematic TKA and the potential weight-bearing MRI might offer call for exploration of low-field weight-bearing MRI to diagnose the problematic TKA. Consequently, the aim of this feasibility study was twofold. First, to compare the diagnostic value of low-field weight-bearing MRI concerning pathologies associated with a problematic TKA with CT and surgical findings during revision surgery. Secondly, to evaluate differences between supine and weight-bearing low-field MRI for patellofemoral parameters after TKA.

## Methods

### Patient selection criteria

**Fig. 1:**
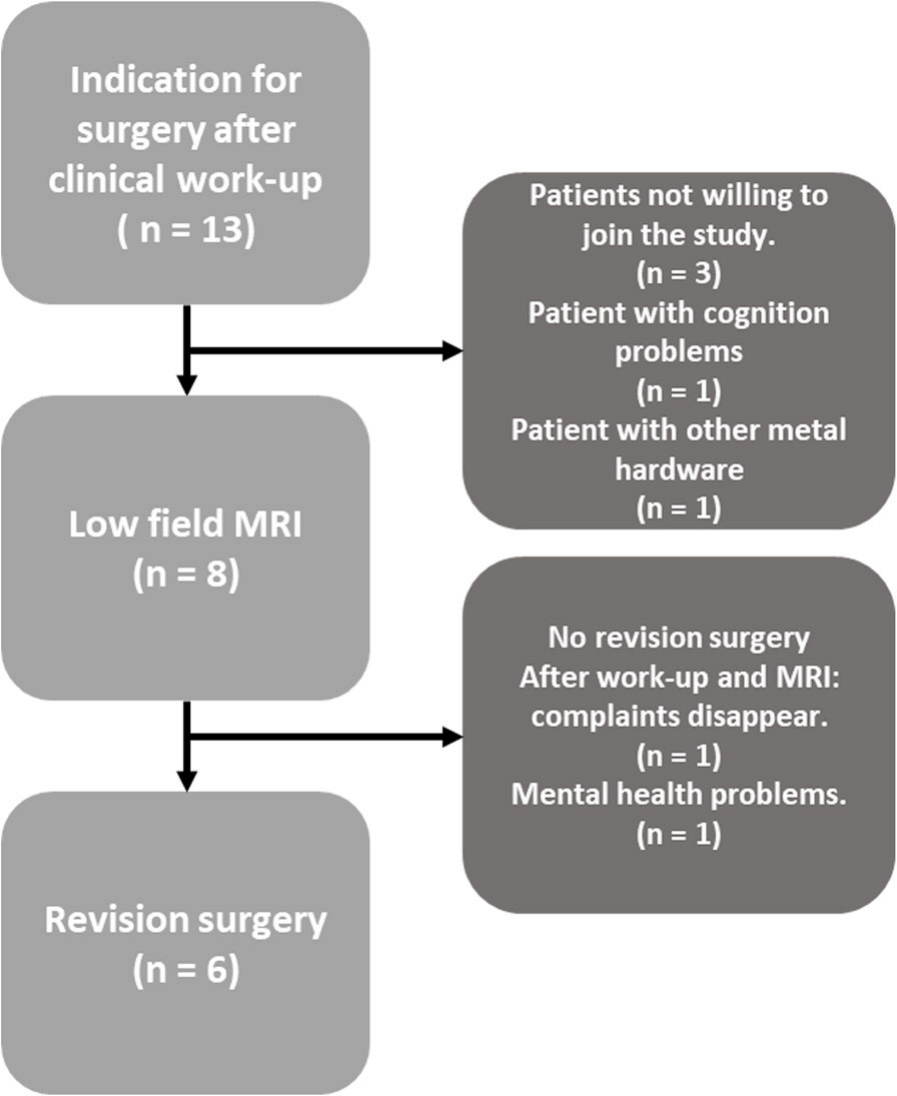
Patients selection flowchart

A prospective feasibility study was conducted between November 2018 and June 2019 and eight patients with a problematic TKA (three male and five females, median age 67 years (range 55–72), two left and six right knees) were consecutively included at OCON Centre for Orthopaedic Surgery (Hengelo, The Netherlands) (Fig. 1). In six out of eight patients the complaints started between the first and third year after TKA. In two patients, the complaints started nine and ten years after TKA. Given there is no available data on the diagnostic value of low-field weight-bearing MRI, a proper sample size calculation could not be conducted. The number of eight patients has been found to be the minimum required in both public and industry-funded pilot and feasibility trials [19]. Inclusion criteria comprised patients dissatisfied after primary TKA (NexGen, posterior stabilized, BiometZimmer) and patients considered eligible for revision surgery based on the standard clinical work-up. Exclusion criteria were a body mass index of over 35 kg/m^2^, other implanted devices that could interact with the magnetic field, and the inability to stand for the duration of the MRI experiment. Informed consent was obtained from all patients.

All patients were scheduled for revision surgery within six months after the low-field MRI experiment. For one of the patients, the complaints disappeared before surgery, while a second patient encountered other health problems (Fig. 1). Therefore, six patients underwent revision surgery, performed by an experienced orthopaedic knee revision surgeon. The revisions were an insert change (two patients, + 4 mm thicker liner), patella resurfacing (one patient), tibia component revision (one patient), and full component revision to a rotating hinge (two patients) implant. During the revision surgery, the causative findings relating to the revision indication were recorded.

### Image acquisition

**Fig. 2:**
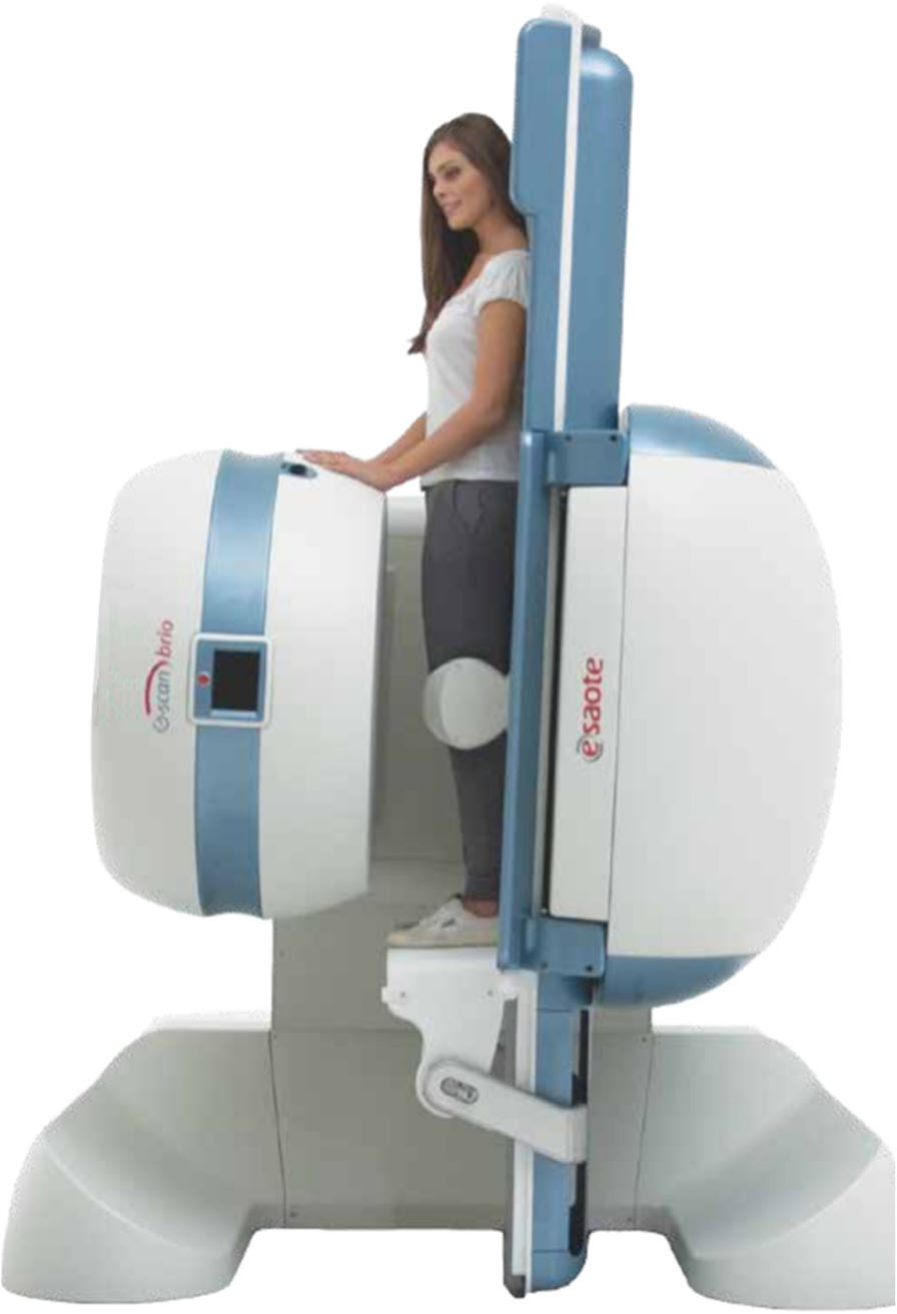
Scanning position in weight-bearing condition in a low-field MRI-scanner. Adapted with permission from Esaote (esaote.com)

Patients were scanned at the University of Twente (Enschede, The Netherlands) on a low-field 0.25 T MRI system (G-scan brio; Esaote SpA, Genova, Italy) in the weight-bearing and supine conditions (Fig. 2), using a dedicated knee coil Spin echo (SE), fast spin-echo (FSE), and X-MAR sequences (based on the view angle tilt (VAT) technique). The sequences used were T1, T2, and PD weighted (TR/TE 1160–7060 ms/12–72 ms) in the sagittal, coronal and transversal directions. Slice thickness was 4 mm, with a gap of 0.4 mm. The field of view was between 200 mm and 260 mm, with an acquisition matrix of either 256 × 256 or 512 × 512. The weight-bearing examinations were performed first, with the patient table at an angle of 81^0^. Both knees were under physiological load during the weight-bearing examination. Thereafter, supine examination was performed. The total duration of the imaging protocol was approximately 30 min (five minutes for positioning and rotation, 12 min for weight-bearing MRI, one minute for rotation and repositioning, 12 min for supine MRI).

### Measurements

**Fig. 3:**
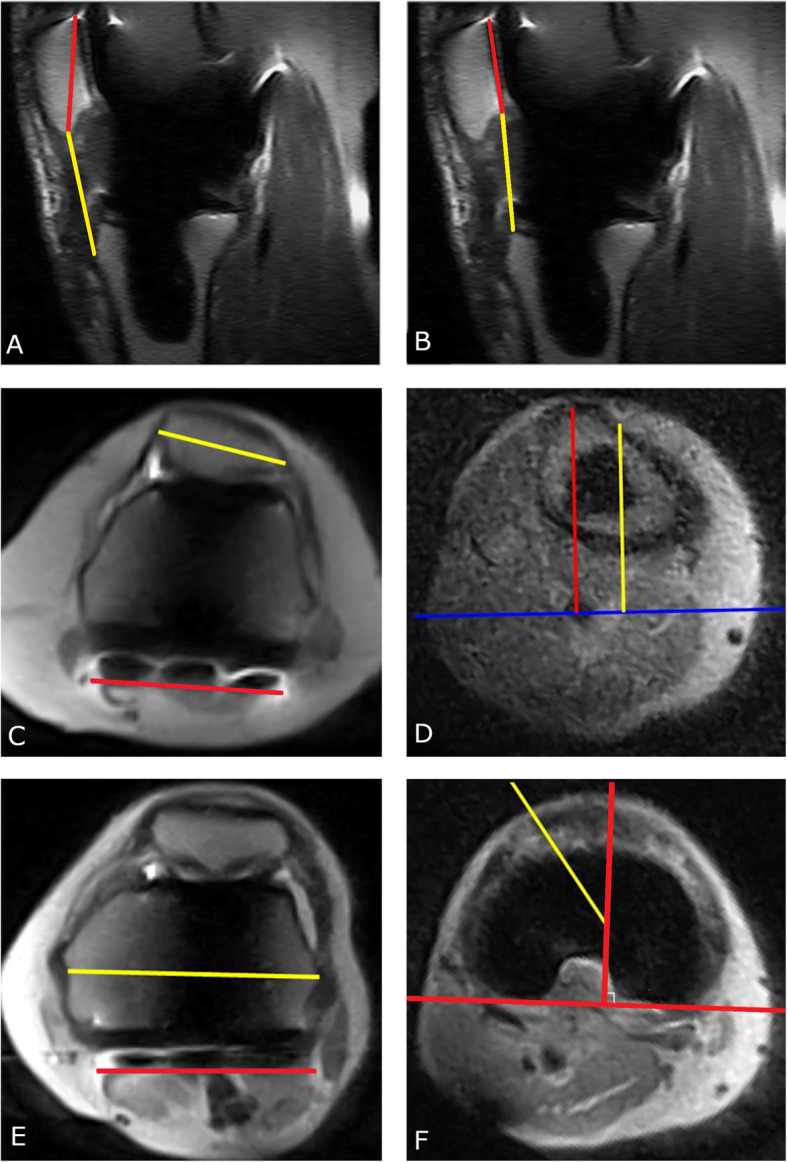
Measuring patellofemoral and rotational alignment parameters. The patellar height was measured with the Insall-Salvati ratio (**a**), the length of the patellar tendon (yellow line) is divided by the diagonal length of the patella (red line), and with the Caton-Deschamps ratio (**b**), the distance between the distal pole of the patella and the tibial plateau (yellow line) is divided by the posterior length of the patella (red line). The patellar tilt angle (**c**) was measured as the angle between the maximum width of the patella (yellow line) and the posterior condylar axis (red line). The tibial tubercle- trochlear groove distance was measured on three levels, at the first level a line through the posterior epicondyle (blue) was drawn, at the second level a line through the deepest point of the trochlear groove (yellow) perpendicular to the posterior epicondyle was drawn, then on the third level a line (red) through the most anterior portion of the tibial tuberosity is drawn (**d**). The distance between the red and yellow line is the TT-TG distance. The femoral component rotation (**e**) is measured on two levels as the angle between the posterior condylar axis (red line) and the surgical transepicondylar axis (yellow line). The tibial component rotation (**f**) is measured on three levels, on the first level the centre of the tibia is determined, then on the second level the centre of the tibia is connected to the top of the tibial tuberosity (yellow line), next the angle between the yellow line and the line perpendicular on the tangent of the tibia plateau of the tibial component (red) is calculated

The MRI scans were assessed by a radiologist with 10 years’ musculoskeletal experience, who was unaware of the clinical diagnosis and findings during surgery. His MRI report described the status of prosthetic fixation and (pathologies of) the surrounding structures, including bone, tendons, ligaments, and muscles. Moreover, patellofemoral alignment parameters and rotational alignment parameters were measured by an imaging expert, who was also kept blind to patient characteristics. The measurements were performed as shown in Fig. 3. The Insall-Salvati ratio (IS, normal value in the native knee 0.8–1.2 [17]) and the Caton-Deschamps ratio (CD, normal value in the native knee 0.6–1.2) were used to evaluate the patellar height [20]. Patellar tilt was evaluated based on the patellar tilt angle (PTA, normal value in the native knee 3^0^–7^0^) [17]. Moreover, the tibial tubercle-trochlear groove distance (TT-TG)) was measured (10–15 mm in the native knee) [21]. The rotational alignment was evaluated through tibial component rotation (TCR) using the Berger angle and via femoral component rotation (FCR) measuring the posterior condylar axis (PCA) [22]. The images were evaluated semi-automatically using Matlab software that was developed in house (R2018a, The Mathworks, Natick, USA). To be able to compare the MRI observations with the clinical diagnosis, the standard clinical work-up results and the surgical findings during revision surgery were extracted from the patients’ medical records. Standard clinical work-up included a diagnosis based on anamnesis, clinical examination, radiological reports made by a musculoskeletal radiologist based on conventional knee radiographs and an indicative CT or other additional investigations, such as stress radiographs or bone scintigraphy.

The study was approved by the Medical Research Ethics Committees of Twente (Netherlands Trial Register: Trial NL7009 (NTR7207). Registered 5 March 2018, https://www.trialregister.nl/trial/7009).

### Analysis

MRI observations were descriptively compared with the diagnosis based on CT results, clinical diagnosis, and findings during surgery. The results of these comparisons were scored in consultation between the radiologist and the image expert. When the diagnoses based on MRI was comparable with CT results, clinical diagnosis or findings during surgery, it was scored as excellent agreement (++). If the diagnoses based on MRI was partly comparable with CT, clinical diagnosis or surgery, it was scored as a moderate agreement (+). When the diagnoses based on MRI was not comparable at all it was scored as no agreement (−-). If CT, clinical findings or surgery were not available the agreement was scored as not applicable (n/a). Based on the comparison of the clinical diagnosis and findings during surgery with the low-field MRI observations, several pathologies were discussed. For the patellofemoral alignment parameters of each patient, weight-bearing and supine values were plotted together with their normal ranges for the native knee, as reported in the literature. Due to the small sample size, statistical paired differences for each of the eight patients’ patellofemoral parameters, in both the weight-bearing MRI and supine MRI conditions, were evaluated utilizing the non-parametric Wilcoxon signed-rank test. The statistical analyses were performed using SPSS (version 25, IBM Corp., Armonk, NY, USA). The level of significance was set at *p* < 0.05.

## Results

In all six patients who underwent revision surgery, the diagnosis based on the clinical work-up was comparable with the findings during surgery. Table 1 describes the results from the clinical work-up i.e. the diagnosis, the low-field MRI observations, and the findings during surgery. As can be seen in Table 1, in the majority of the cases (four out of six), low-field MRI observations were roughly the same as the findings during surgery. For six of the eight patients, the diagnosis based on the clinical work-up was in line with low-field MRI observations. Interestingly, all MRI observations were comparable with the CT results. However, the additional weight-bearing MRI did not reveal additional information regarding the diagnosis based on low-field MRI.

**Table 1:** Clinical diagnosis with additional imaging results + low-field MRI observations + findings during surgery per patient

	***Clinical work-up***				***Clinical diagnosis***	***Low-field MRI***	***Findings during surgery***	***Agreement***		
Patient	Anamnesis	Conventionalradiographs	CT	Other				D & MRI	OR & MRI	CT & MRI
1	Instability anddiffuse pain	Negative	Negative	Varus & valgus stress films, positive	Collateral laxity	Negative	Collateral laxity	–	–	++
2	Pain anterolateral and swelling	Negative	9^0^ internal rotation of the tibial component	Varus & valgus stress films, positive	Collateral laxity, no clinical malposition	11^0^ internal tibial component rotation	Collateral laxity	–	–	++
3	Instability and pain anterolateral	Negative	4^0^ internal rotation of the femoral component	Valgus stress films,positive	Malalignment of the femoral component	3^0^ internal rotation of the femoral component	Malalignment of the femoral component	++	++	++
4	Pain anterolateral, medial and swelling	Negative	6^0^ internal rotation of the tibial component	Varus & valgus stress films, positive	Malalignment and asymmetric laxity	5^0^ internal tibial component rotation	Malalignment of the tibial component	++	++	++
5	Anteriorknee pain	Tibial component loosening	Lucency around tibial component suspected for loosening	Negative punction and lab	Tibial component loosening	Effusion around the medial side of the tibial component and the MCL	Partial tibial component loosening.	+	+	+
6	Medial knee pain	Tibial component loosening	Lucency around tibial stem suspected for loosening	Negative punction and lab	Tibial component loosening	Effusion around the tibial stem	n/a	+	n/a	+
7	Diffuse painand swelling	Negative	Lucency around the tibial component. Possible early loosening	Not applicable	Early tibial component loosening	Joint effusion	n/a	+	n/a	+
8	Pain anterolateral and during stair climbing	Negative	n/a	Bone scintigraphy showing patellofemoral activity	Patellofemoral arthroses	Patellofemoral arthroses	Patellofemoral arthroses	++	++	n/a

*D = diagnosis, OR = findings during surgery, ++ excellent agreement, + moderate agreement, −- no agreement, n/a. not applicable*

**Fig. 4:**
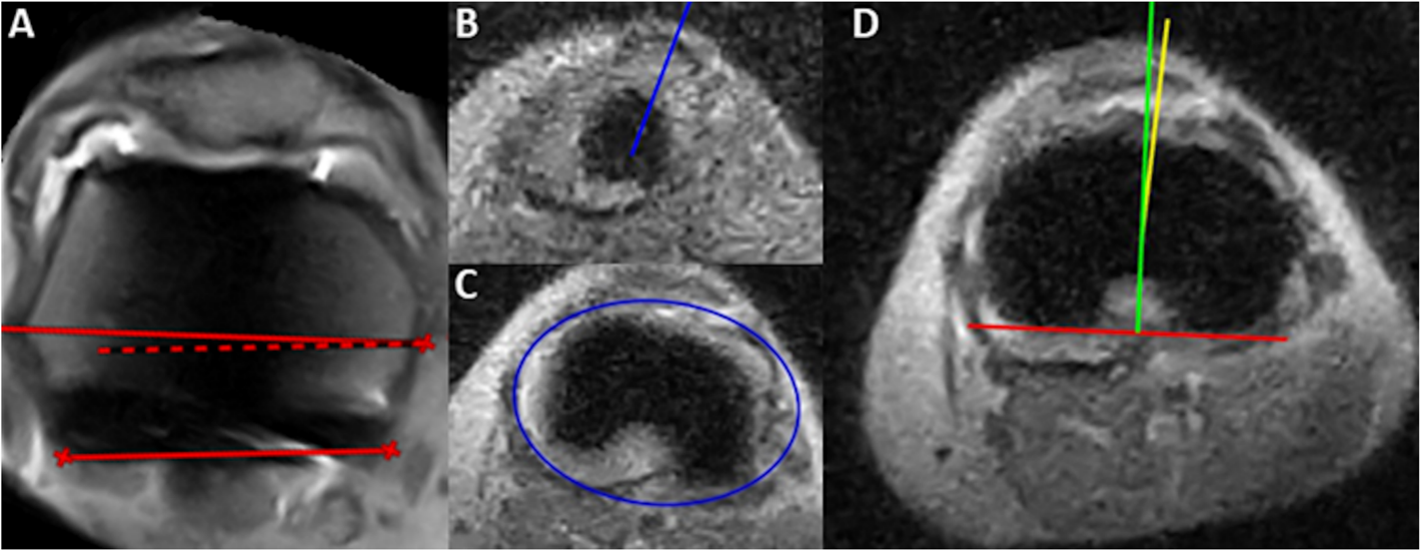
Rototional malalignment of the TKA. **a**) shows 3.3^0^ of internal rotation of the femoral component of patient 3, measured as the angle between the posterior condylar axis and the surgical transepicondylar axis. **b**-**d**) show 4.4^0^ tibial component rotation, measured in accordance with the Berger protocol, wherefore **b** shows the top of the tibia tuberosity, which is connected with the centre of the tibia determined in **c**. In **d**, the angle between the yellow line and the green line perpendicular on the tangent of the tibia plateau of the tibial component (red) was calculated as 4.4^0^ tibial component rotation

**Fig. 5:**
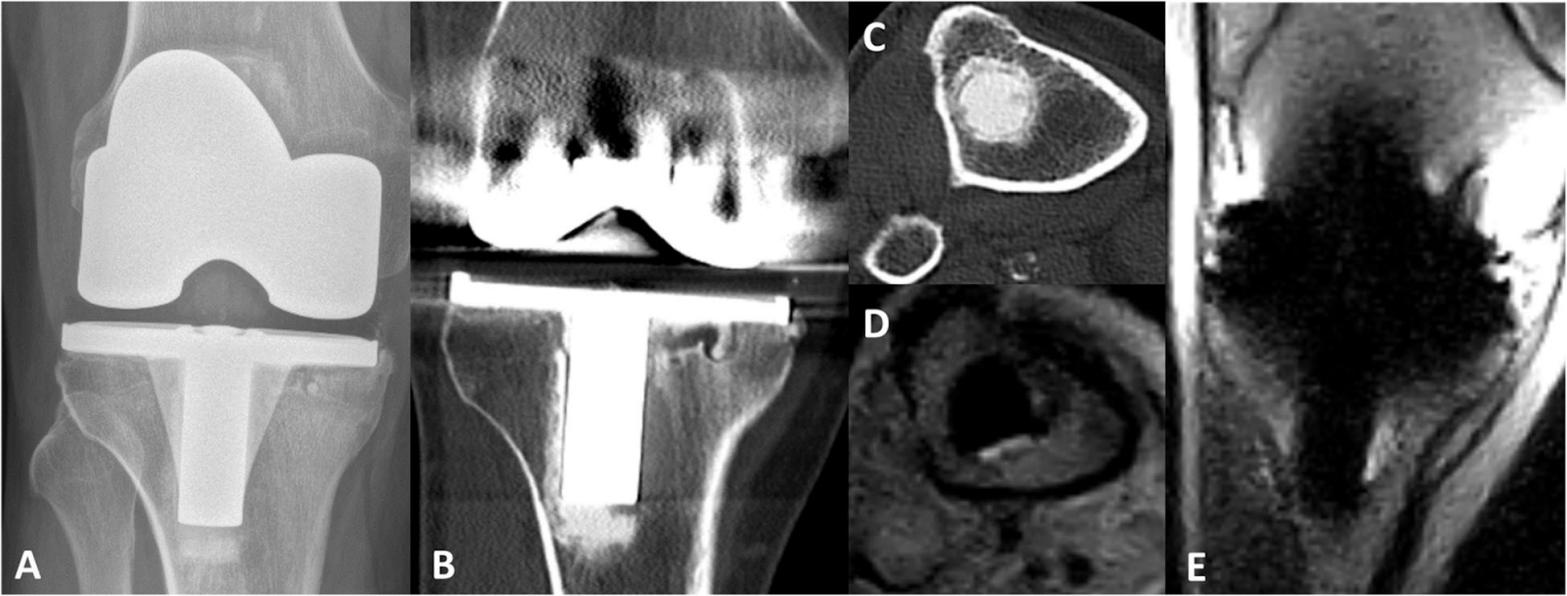
Loosening of the tibial component. The conventional radiograph (**a**) of patient 6 shows tibial component loosening around the medial plate and stem. CT (**b** and **c**) and low-field MRI (**d** and **e**) show images of the same knee, where images **c** and **d** are the transversal views of **b** and **e** at the most distial point of the tibial stem. The CT shows lucency (**b**-**c**) and MRI effusion (**d**-**e**) around the tibial stem, which are elements suspected of loosening the tibial component

**Fig. 6:**
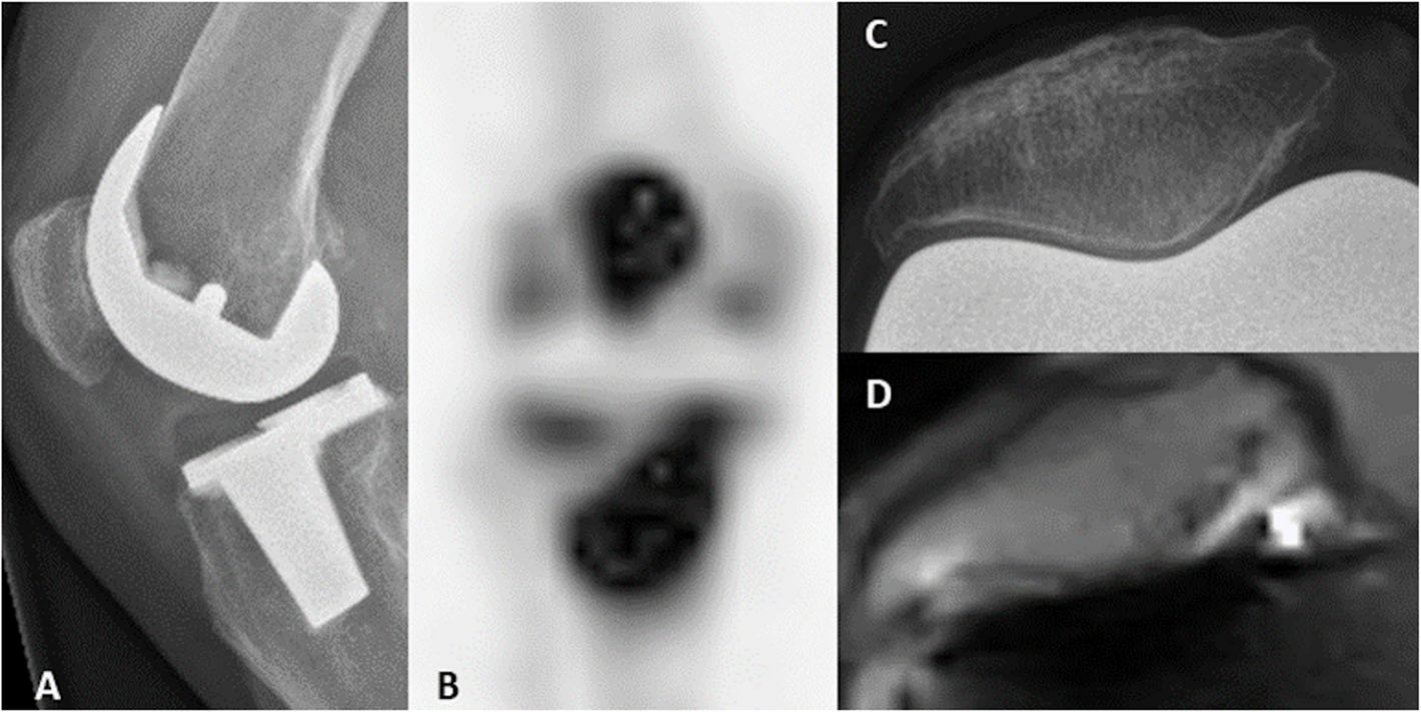
Patellofemoral arthrosis. The conventional radiograph (**a**) of patient 8, together with the bone scintigraphy (**b**), the additional patellofemoral radiograph (**c**) and the low-field MRI (**d**). Except for the conventional radiographs (**a**), all other images **b**-**d**) show patellofemoral arthrosis

Malalignment was measured and confirmed with CT in three cases. Figures 4 shows a typical example of prosthetic component malalignment measured on the low-field MRI of two different patients (patients three and four). Prosthetic loosening (tibial component) was the clinical diagnosis for patient six. Figure 5 shows the low-field MRI, which showed high signal on the T2-weighted images surrounding the tibial stem. Over time, the patient indicated that complaints had considerably reduced and the revision surgery was consequently cancelled, making it impossible to compare the data with the findings during surgery. Patellofemoral arthrosis was found on the MRI of patient eight (Fig. 6), which was analogous to the clinical diagnosis and surgical findings. Signs of laxity could not be diagnosed based on low-field MRI (case one and two). In case of ligament instability, stress radiographs remains the superior diagnostic modality.

**Fig. 7:**
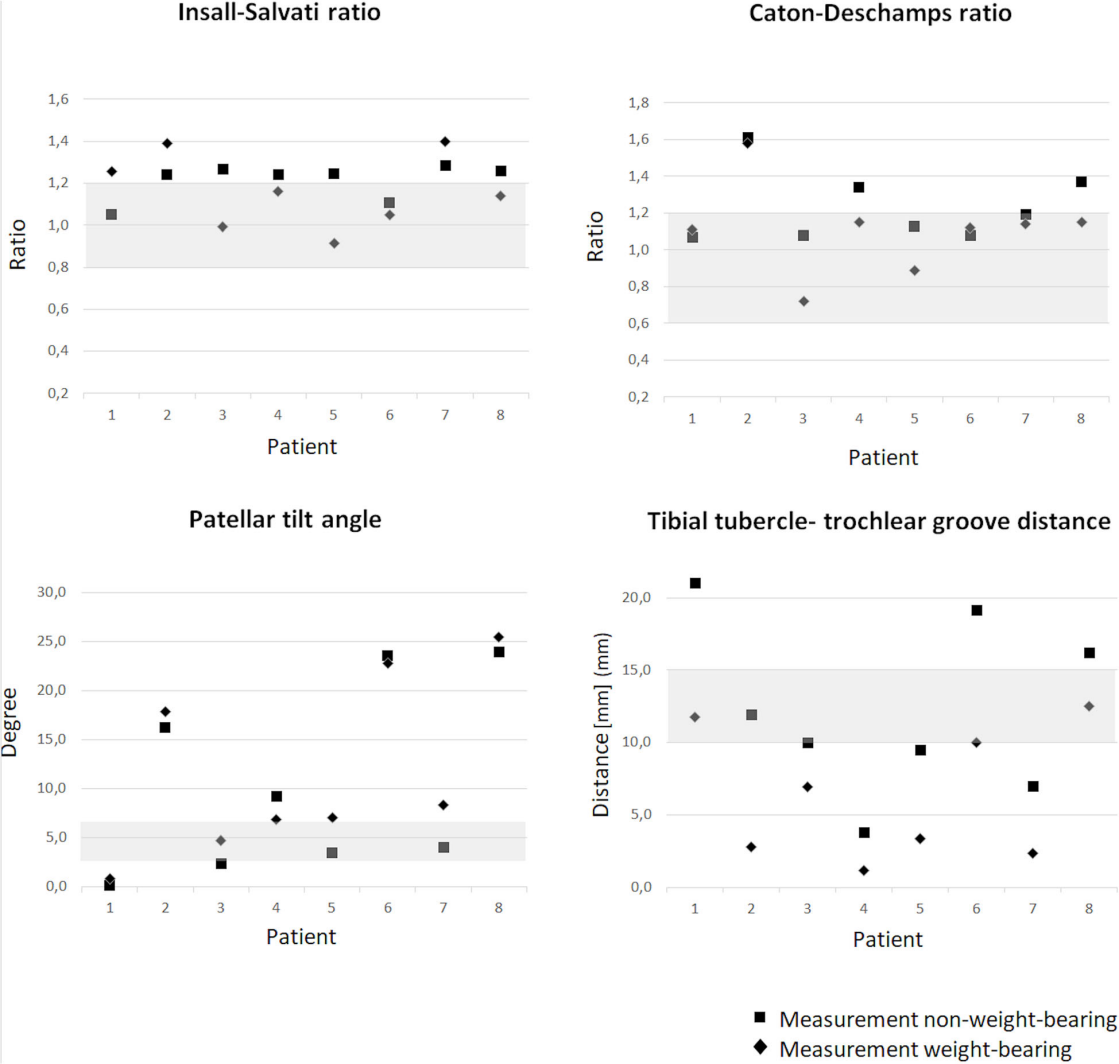
Results of the patellofemoral measurements of eight patients with a problematic TKA, scanned in weight-bearing and non-weight-bearing conditions using low-field MRI to measure the IS and CD ratios, the PTA and the TT-TG distance. The grey areas are the ranges given in the literature for the native knee: Insall-Salvati ratio (0.8–1.2) [17], Caton-Deschamps ratio (0.6–1.2) [20], Patellar tilt angle (3^0^–7^0^) [17], tibial tubercle-trochlear groove distance (10–15 mm) [21]

For all patients, patellofemoral parameters were measured in weight-bearing and supine conditions. Figure 7 illustrates the patellofemoral parameters per patient, per condition. Interestingly, the TT-TG distance significantly decreased in weight-bearing condition (*p* = 0.012). For the other parameters, no significant differences between supine and weight-bearing conditions were found (IS (*p* = 0.575), CD (*p* = 0.068), PTA (*p* = 0.161)). However, there seemed to be a trend in the decrease of CD and PTA in the weight-bearing condition.

## Discussion

Identifying the underlying pathologies that cause a problematic TKA is often challenging. This is the first study attempting to explore the diagnostic potential of low-field weight-bearing MRI for imaging pathologies associated with a problematic TKA and compare MRI findings with clinical diagnosis, CT findings and surgical findings. In six out of the eight cases included in this study, the MRI observations were in line with the diagnosis based on the clinical work-up, and in four out of six cases, the MRI observations of malalignment, suspected loosening, and patellofemoral arthrosis were confirmed with findings during revision surgery. Only collateral laxity could not be confirmed with low-field MRI. Importantly, all MRI observations were comparable with CT or scintigraphy results. Weight-bearing MRI significantly decreased TT-TG distance measurements when compared to supine MRI. In addition, the other patellofemoral parameters showed a decreasing trend when measured in the weight-bearing condition. However, the added value of weight-bearing low-field MRI to evaluate the problematic knee could not be proven yet. Based on our study results, low-field MRI shows a comparable diagnostic value to CT regarding evaluation of the problematic TKA, but currently cannot replace the entire clinical work-up and solely diagnose all pathologies associated with the problematic TKA.

In the cases described in our study, rotational component malalignment could be diagnosed with low-field MRI. As demonstrated in the extant literature, rotational malalignment has been possible to diagnosed by means of high-field MRI [23, 24]. In this study, CT or MRI results for component malalignment were not always supported by the clinical diagnosis. This was due to the fact that not all patients with measured component malalignment had clinical complaints related to malalignment. During the evaluation of synovitis, which is related to aseptic loosening [25–27], the assessed low-field MRI images did show increased signal in T2 scans surrounding the tibial component, which has been associated with aseptic loosening in several high-field MRI studies [25–27]. However, as only one of these patients with observations of synovitis on low-field MRI underwent surgery, the clinical evidence is scarce, and more cases are needed to reach a definitive conclusion. Patellofemoral arthrosis could also be visualised with low-field MRI, as the observations were in line with the clinical diagnosis based on the bone scintigraphy and the findings during surgery. Pathologies only causing laxity could not be diagnosed based on the low-field MRI scans and were only visible on the stress radiographs. It was expected that low-field MRI would provide additional diagnostic information concerning soft-tissue problems, as MRI is the superior imaging modality to diagnose these kind of problems in the native knee [10]. Unfortunately, this could not be confirmed in the current study due to the fact that no patients with soft tissue problems, such as a tendinopathy, could be included. Results show that it is possible with low-field MRI to image the soft tissue structures surrounding the prosthetic components, which made it of potential added value when soft tissue problems are present.

When comparing the results of weight-bearing versus supine MRI, as expected, a significant decrease of the TT-TG distance was found in the weight-bearing condition. This result is in line with findings in the native knee [28] and satisfied knee after TKA, and can be explained by quadriceps loading [29]. Moreover, the results suggest a decreasing trend in patellofemoral parameters between the weight-bearing and supine conditions for the CD and the PTA. When evaluating all four patellofemoral measurements, there is a notable deviation between the measurements performed in this study and the normal values in the native knee [17, 20, 21]. However, the clinical relevance of these differences is unknown; as there are no reference values for patellofemoral parameters after TKA, no firm conclusions can be drawn between the measured patellofemoral parameters and the patients’ complaints yet. In the future, measurement and collection of patellofemoral parameters after TKA would be a possible area of study. When more data is available, normal values can be determined and perhaps patellofemoral measurement outliers in the weight-bearing condition can be related to the cause of the problematic TKA, thereby improving diagnostics.

Although the current study is the first to explore the diagnostic feasibility of low-field MRI regarding pathologies associated with the problematic knee, there are some obvious limitations. First, given the explorative character of the current study the sample size was kept limited and heterogeneous to represent the variety of reasons for the problematic TKA. If considerable differences would exist between patellofemoral measurements based on weight-bearing MRI and supine MRI, they would have been found even with a small sample size. However, in this feasibility study it is less important whether the difference found is statistically significant but much more about whether it could be of clinically relevance to the patient. Although small differences were found between patellofemoral parameters in weight-bearing and supine conditions, differences of clinical relevance were not perceived. Therefore, to be more certain about the diagnostic value of low-field MRI and the added value of weight-bearing MRI, more patients need to be scanned. The current study reveals an estimate of variability between the weight-bearing and supine positions for patellofemoral parameters, which can be used to conduct proper sample size calculations to set up clinically relevant studies in future research. Second, as radiologists are trained to assess high-field MRI scans, it was more difficult to evaluate images made on a lower field strength. Soft tissue structures, such as the popliteus tendon and the semi-membranous tendon, which are close to the posterior part of the prosthetic components, were especially challenging to distinguish. This is likely caused mainly by the reduced signal-to-noise ratio (SNR) of low-field MRI, and partly due to susceptibility artefacts caused by the TKA. Since, malalignment of the tibial component affects posterior tendon tension [30], and MRI (in contrast to CT) offers the ability to image soft tissue, it would be beneficial if those structures can be visualised.

In clinical practice, a CT scan is often made when additional imaging is needed. In this study, diagnostic findings considering the problematic TKA based on the low-field MRI were interchangeable with the diagnostic findings based on CT. When comparing these two imaging modalities low-field MRI does not use any ionizing radiation, and offers the possibility to image soft tissues surrounding the prosthetic components. Since soft tissue problems are difficult to diagnose with CT, it can be expected that if soft tissue problems are present low-field MRI might make a difference. Moreover, when comparing purchasing and maintenance costs with high-field MRI, low-field MRI is just as CT by a rough estimation 3 times less expensive [31]. Hence, from a cost perspective, low-field MRI may be a realistic competitor for CT. These factors made it relevant to study whether low-field MRI could be used as a cost- efficient and effective alternative in diagnosing problems around a problematic TKA.

Currently, there is not one imaging technique capable of differential diagnosis in the problematic knee after TKA. This study focused on the diagnostic value of low-field MRI. However, when evaluating the standard clinical work-up, it is remarkable that the conventional radiographs were of added value in only two out of the eight cases. In all other cases, additional imaging by CT, bone scintigraphy or stress radiographs was needed to further diagnose the problematic TKA. Low-field MRI is an addition to the diagnostic arsenal. Low-field MRI is capable of simultaneously diagnosing different pathologies, such as malalignment, loosening and patellofemoral arthrosis. In our study, low-field MRI could not diagnose laxity and other pathologies such as soft tissue problems. Infection was not present in our population and, therefore, the efficacy of low-field MRI on these subjects remains unknown. Further research is warranted to determine the clinical and cost-effective value of low-field MRI among the current imaging arsenal in patients who are dissatisfied with their TKA.

## Conclusions

This feasibility study showed the potential of low-field MRI to image pathologies associated with a problematic total knee arthroplasty. The, diagnoses based on low-field MRI were comparable to the diagnoses based on CT. Our hypothesis of the added value of weight-bearing MRI to diagnose patellofemoral problems associated after primary TKA could not be supported in this feasibility study.

## Abbreviations

CD: Caton-Deschamps ratio
FCR: Femoral component rotation
FSE: Fast spin-echo
IS: Insall-Salvati ratio
PTA: Patellar tilt angle
PCA: Posterior condylar axis
SNR: Signal-to-noise ratio
SE: Spin echo
TCR: Tibial component rotation
TKA: Total knee arthroplasty
TT-TG: Tibial tubercle-trochlear groove distance
VAT: View angle tilt
